# Ethnic minority health in Vietnam: a review exposing horizontal inequity

**DOI:** 10.3402/gha.v6i0.19803

**Published:** 2013-03-04

**Authors:** Mats Målqvist, Dinh Thi Phuong Hoa, Nguyen Thanh Liem, Anna Thorson, Sarah Thomsen

**Affiliations:** 1 International Maternal and Child Health (IMCH), Department of Women's and Children's Health, Uppsala University, Uppsala, Sweden; 2 Hanoi School of Public Health, Hanoi, Vietnam; 3 Research Institute for Child Health (RICH), National Hospital of Pediatrics, Hanoi, Vietnam; 4 Division of Global Health (IHCAR), Department of Public Health, Karolinska Institutet, Solna, Sweden

**Keywords:** ethnic minorities, Vietnam, inequity, policy, maternal health, child health, HIV, nutrition

## Abstract

**Background:**

Equity in health is a pressing concern and reaching disadvantaged populations is necessary to close the inequity gap. To date, the discourse has predominately focussed on reaching the poor. At the same time and in addition to wealth, other structural determinants that influence health outcomes exist, one of which is ethnicity. Inequities based on group belongings are recognised as ‘horizontal’, as opposed to the more commonly used notion of ‘vertical’ inequity based on individual characteristics.

**Objective:**

The aim of the present review is to highlight ethnicity as a source of horizontal inequity in health and to expose mechanisms that cause and maintain this inequity in Vietnam.

**Design:**

Through a systematic search of available academic and grey literature, 49 publications were selected for review. Information was extracted on: a) quantitative measures of health inequities based on ethnicity and b) qualitative descriptions explaining potential reasons for ethnicity-based health inequities.

**Results:**

Five main areas were identified: health-care-seeking and utilization, maternal and child health, nutrition, infectious diseases, and oral health and hygiene. Evidence suggests the presence of severe health inequity in health along ethnic lines in all these areas. Research evidence also offers explanations derived from both external and internal group dynamics to this inequity. It is reported that government policies and programs appear to be lacking in culturally adaptation and sensitivity, and examples of bad attitudes and discrimination from health staff toward minority persons were identified. In addition, traditions and patriarchal structures within ethnic minority groups were seen to contribute to the maintenance of harmful health behaviors within these groups.

**Conclusion:**

Better understandings of the scope and pathways of horizontal inequities are required to address ethnic inequities in health. Awareness of ethnicity as a determinant of health, not only as a covariate of poverty or living area, needs to be improved, and research needs to be designed with this in mind.

Inequity in health is a major challenge for health care planners and policy makers all over the world. Whether it is in countries, such as China or Vietnam, both with booming economies, or in countries struggling with budget deficits and reductions in government spending, the gap in health status between the disadvantaged populations and the most favored is increasing (1–3). This development needs to be addressed, and effective strategies to close the gap are needed. There are a large number of structural factors, for example, household economic status, gender, ethnicity, and caste that could cause health inequities. Furthermore, these are often intertwined with each other (4, 5). Although there is evidence of inequity being based on factors independent of personal or household wealth, inequities have primarily been addressed in economic terms. In previous studies from Vietnam, for example, we have shown that there is an increased risk of neonatal mortality in ethnic minority groups, regardless of education or household economic status (6, 7), and that women have fewer possibilities than men to receive a diagnosis of tuberculosis (TB), depending on a combination of societal and health care provider-related factors (8).

For many years, Vietnam has exhibited good results in health outcomes, providing an example of a low-income country that has succeeded in its public health efforts despite lack of resources (9). The relative success of Vietnam's health sector has often been attributed to a comprehensive public health system covering the whole population with more than 10,000 commune health stations and 600 district hospitals (9). Large-scale public health campaigns like the Expanded Program of Immunization (10) and measles vaccination drives have also been successful, contributing to improved public health (11). This development has, however, not been without difficulties. In particular since the reform period (*doi moi*), there have been a number of health reforms trying to manage the transition from a government financed health system to a more market oriented approach (12). The multitude of uncontrolled private health care providers or drug sellers pose a special challenge. Several studies have shown how, for example, women are more likely to use under qualified private providers or drug sellers (13, 14), which in turn may delay adequate diagnosis (15). Apart from trying to find a feasible financing system for the health system, a special focus has been on the poorer segments of the population, both through the National Target Programs on Health in the 1990s and later with the National Health Care Fund for the Poor (HCFP), launched in 2003 (16). Even so, there are many examples of inequities in health based on economic status, and with the present rapid economic and social development, the equity gap is likely to increase (17, 18).

In existing reports on major surveys and censuses, there is a severe lack of analysis based on ethnicity. The Demographic and Health Survey (DHS) 2002 and the AIDS Indicator Survey (AIS) 2005, for example, have recorded ethnicity during data collection but not presented ethnicity as an independent variable when presenting results (19, 20). In the Population and Housing Census of 2009, only crude infant mortality rate for a selection of ethnic minority groups is presented (21). In the Vietnam Household Living Standard Survey of 2006 and 2008, ethnicity is included as an independent variable but no further analysis other than crude figures are presented in the reports from the General Statistics Office (GSO) (22, 23). There is no reliable registry data available, due to the lack of a trustworthy vital registration system (24, 25).

There are 54 ethnic groups in Vietnam, with Kinh constituting the majority of the population (84%) (26). Some ethnic groups are small, with less than 1,000 members, and apart from the Hoa (Chinese) group, ethnic minorities are disproportionately poor and generally live in more remote locations (27, 28). Historically, the ethnic minorities of Vietnam have had an ambiguous role in the construction of the Vietnamese nation state. Although they are regarded as a national treasure, with their rich cultural diversity, ethnic minorities have been subject to far-reaching reform programs, aiming at raising living standards, which sometimes resemble assimilation attempts (29, 30). Efforts have been made from the Ministry of Health to target ethnic minority groups through the identification of prioritized communes in a development policy labeled Programme 135. This policy aims to increase living standards in the selected communes and includes benefits from health care free of charge for the communes’ entire population (31). However, this targeting is primarily based on economic evaluation and not with culture and tradition as its main concern (32), while at the same time ethnic minority culture is displayed as a national asset (29). Some scholars contribute this contradictory official standpoint to the common perception of ethnic minorities among the Vietnamese majority (29, 33). Ethnic minorities are depicted, in official development reports as well as in national media, as ‘backward’ and ‘deficient’ (29, 30), while at the same time considered to be representing the rich and colorful culture of Vietnam at festivals and in tourist commercials (33). The disadvantaged position of ethnic minorities have mostly coincided with their low socioeconomic status, and it is not until recently that a focus on ethnicity as such has begun to be considered in reports and literature (29). It is also commonly acknowledged that other factors besides economic status direct health outcomes, but studies explaining the ‘how’ and ‘why’ of inequities in health are scarce. Therefore, the research aim of the present review is to highlight ethnicity as an important predictor in public health over and above household economic status and education. The specific objectives are to examine the existing evidence of explanations and causes of ethnic inequity in health and to summarize findings of the magnitude of related to ethnicity in Vietnam through a systematic review.

## Methods

This review was set up as a systematic review in order to get a comprehensive overview of evidence in relation to ethnic minority health in Vietnam. It has, however, been argued that the narrow focus of the systematic review when it comes to analysis does not allow for the full coverage of a topic (34). Therefore, the analysis of the included publications has used narrative methods extracting data relevant to the aim.

### Study inclusion/exclusion criteria

Studies published in academic journals and the grey literature from 1990 to 2011 was included for review. Only original research presented in English was considered, and all types of study designs were included. Since the aim of the study was to review the academic literature about health among ethnic minorities in Vietnam, we excluded all articles where the main study population was living outside of Vietnam and studies not relating to health issues. Quantitative studies that had no comparisons between ethnic groups were also excluded. Qualitative studies that aimed to explain differential health outcomes between ethnic minority and majority populations were included. Therefore, we discarded qualitative studies that did not explicitly aim to explain determinants of inequity. Reviews, merely citing previous research, were excluded, while reports containing secondary data analysis were included (Table 1). No preset study protocol was used or registered for this systematic review.

**Table 1:** Inclusion and exclusion criteria for studies

Inclusion criteria	Exclusion criteria
• Studies published in English	• Studies in other languages than English
• Studies published from 1990 up until September 2011	• Studies with no comparison between ethnic groups
• Qualitative studies aiming to explain differences in health between ethnic groups due to cultural and societal factors	• Studies with study population outside Vietnam
	• Reviews
• Quantitative studies measuring the impact of ethnicity on health outcomes	• Studies with no health related outcomes

### Search strategy and study selection

**Fig. 1:**
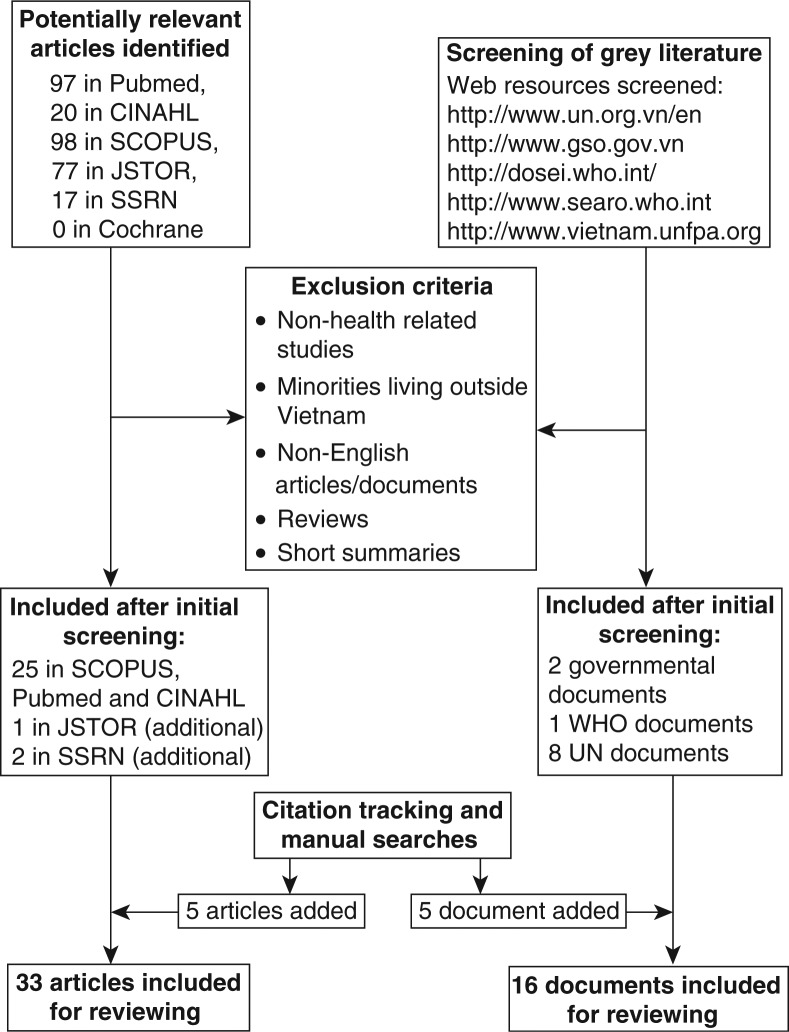
Flow chart.

An initial literature search in relevant databases was performed. CINAHL and PubMed were searched using the search string [ethnic* AND minor* AND Vietnam]. JSTOR, SSRN, and SCOPUS (including full coverage of MEDLINE, EMBASE, and ISI Web of Knowledge) were searched using the same search string with the addition of [AND health], with the further addition of [NOT veteran NOT America*] in JSTOR. Eligibility assessment based on inclusion and exclusion criteria was performed by the first author. Citation tracking and cross-reference checking were performed after eligibility assessment on the articles included and relevant bibliographic searches were performed. Grey literature relating to ethnic minority health was identified through web searches at internet sites of the major governmental and non-governmental institutions and through citation tracking and manual searches (Fig. 1).

### Data analysis

The analysis was conducted in three steps: (1) The first author read through all titles and abstracts of articles included by the search strings using a pre-determined focus on health and ethnicity. Articles fulfilling inclusion criteria were kept and reviewed in full. (2) We categorized the main themes that emerged from the included articles, identifying five main areas: health-care-seeking and utilization, maternal and child health, infectious diseases, nutrition, and hygiene and oral health. (3) We extracted information on: a) quantitative measures addressing inequity in health outcomes based on ethnicity; and b) qualitative descriptions explaining potential reasons for ethnic-based inequities. No meta-analyses of the material were performed due to the small number of identified studies, and thus no summary measures were reported. Results are presented as a narrative.

## Results

Thirty-three articles from peer-reviewed scientific journals and 16 documents from the grey literature, including two governmental reports were included for further reviewing (Fig. 1). After an initial reading, the articles were grouped into five areas: health-care-seeking and utilization, maternal and child health, nutrition, infectious diseases (including HIV/TB, malaria, and neglected tropical diseases), and oral health and hygiene.

### Healthcare seeking and utilization

The first area is an overarching theme dealing with health care seeking and utilization. In general, we found quite a lot of research focusing on this domain since it is an important aspect of health care delivery. However, for this review, eight peer-reviewed articles and four governmental publications were found that had a primary focus on this issue and met the inclusion criteria (Table 2).

**Table 2:** Health care seeking and utilization

Author(s)	Title	Publication	Year	Main results	Study design and sample	The ethnicity variable
Castel P. (36)	Vietnam Health Insurance: Use of Health Care Services by the Poor Efficiency and Equity Issues in the Province of Kon Tum.	Working papers series, Posted at SSRN April 6, 2011.	2011	Less expenditure on ethnic minorities (EM) compared to the non-poor, thus an equity problem in the provision of health care services. All received more expensive treatments when referred to hospital except EM. EM get less blood, fewer lab exams and less surgery (minor and major). EM mothers do not search particular health care support during pregnancy and delivery.	Comparison of individual's health insurance data of the Provincial Social Security Health Insurance and information collected for claim control	Kinh vs. Minority
Committee for Ethnic Minorities/UNICEF. (31)	Reaching out for change: A qualitative assessment of government health care and education policies affecting the women and children of ethnic minorities.	Available at: www.unicef.org/vietnam	2003	Low awareness among ethnic minority groups about governmental programs was a major obstacle for policy implementation.	Observations, group discussions and in-depth and informal interviews	Ethnic minorities in priority communes.
Hong TK, Dibley MJ, Tuan T. (41)	Factors affecting utilization of health care services by mothers of children ill with diarrhea in rural Vietnam.	Southeast Asian J Trop Med Public Health. 2003 Mar: 34 (1):187–98.	2003	Low use of ORS among EM. EM less likely to seek care for children with diarrhea (OR 2.32 CI 1.08–5.00, adj for education, economy and disease severity). EM reported low levels of satisfaction with their local medical services.	Cross-sectional household survey between Nov 1998 and Jan 1999 in three provinces in the south. 1632 women with 1935 children under 5	Kinh vs. Minority
Malqvist M, Nga NT, Eriksson L, Wallin L, Hoa DP, Persson LA. (6)	Ethnic inequity in neonatal survival: a case-referent study in northern Vietnam.	Acta Paediatr. 2011 Mar: 100 (3):340–6.	2011	Increased risk of neonatal mortality for ethnic minority mothers. The elevated risk was maintained even if the EM mothers went to hospital to deliver or if they attended ANC.	Case-referent study including 183 neonatal death cases and 599 referents	Kinh vs. Minority
Ministry of Health and Health Partnership Group. (39)	Joint annual health review 2010 – Vietnam's health system on the threshold of the five-year plan 2011–2015.	Available at http://www.jahr.org.vn	2010	Lower admission to inpatient care for ethnic minorities than for Kinh majority. Actively support ethnic minority students in medical training.	Vietnam Household and Living Standards Survey (VHLSS) 2002, 2004 and 2006	Delta/urban areas vs. mountains/ethnic minority areas
Ministry of Health and Health Partnership Group. (37)	Joint annual health review 2009 – Human resources for health.	Available at http://www.jahr.org.vn	2009	Few health workers from ethnic minorities.	Cross-sectional survey	Kinh vs. Minority
Oosterhoff P, White J, Huong NT. (44)	Family health consequences of modernisation programmes in Black Thai communities.	Cult Health Sex. 2011 Apr 1.	2011	Imposed governmental policies promoting patrilocality and health facility delivery resulted in increased vulnerability to HIV among Thai women. Highlighting the importance of policy-makers attempting to understand cultural institutions and their social function in their specific context.	Focus Group Discussions (FDGs) with men and women separately of mixed ages. In-depth interviews with 84 Thai women	Black Thai community in Dien Bien province
Rheinlander T, Samuelsen H, Dalsgaard A, Konradsen F. (42)	Perspectives on child diarrhoea management and health service use among ethnic minority caregivers in Vietnam.	BMC Public Health. 2011 Sep 6; 11 (1):690	2011	Several obstacles for EM caregivers to seek health services were identified, including gender roles, long travelling distances, concerns about indirect costs and disrespectful treatment.	In-depth interviews of 43 caregivers, 3 Focus Group Discussions (FGDs) and 2 weeks of participatory observations at two Communal Health Stations	Four EMGs in Lao Cai province
Teerawichitchainan B, Phillips JF. (40)	Ethnic differentials in parental health seeking for childhood illness in Vietnam.	Soc Sci Med. 2008 Mar: 66 (5):1118–30.	2008	EM mothers less likely to seek care when children were sick. EM parents less frequently reported illness.	VNHS 2001	Kinh/Chinese vs. Minority
Toan NV, Trong LN, Höjer B, Persson LA. (35)	Public health services use in a mountainous area, Vietnam: implications for health for policy.	Scand J Public Health. 2002: 30 (2):86–93.	2002	EM used public health services less frequently than the majority group (24% vs. 43%). Distance to health facilities was not associated with ethnicity. Ethnic minority people reported more severe diseases (p=0.03). The under-use of health services by ethnic minorities was observed only when distance to the services was far. Failure of fee exemption; almost all EM (98%) had paid for services even though they should have been exempted.	Cross-sectional using four week diary and structured interview form. 1452 individuals from 300 households in three communes in northern Vietnam	Kinh vs. Minority
UNICEF Vietnam and Dien Bien Provincial People's Committee. (43)	An analysis of the situation of children in Dien Bien.	Available at: http://www.un.org.vn/en/	2010	Ethnic minority mothers utilize healthcare services to a lesser extent. Explanations suggested are distance and cost, language barriers, the maintenance of traditional health care beliefs and a lack of knowledge.	FGDs and observational study	EM in Dien Bien Provence
Wagstaff A. (38)	Estimating health insurance impacts under unobserved heterogeneity: the case of Vietnam's health care fund for the poor.	Health Econ. 2010 Feb: 19 (2):189–208	2010	EM less covered by Health Care Fund for the Poor (HCFP) unless they are poor even though they are eligible.	Official data from Vietnam Social Security (VSS) and household survey data from VHLSS 2002, 2004 and 2006, triple differencing with matching	Ethnic minorities eligible for HCFP

Generally, ethnic minorities are poorer and live in more remote areas than the Kinh majority. Thus, efforts directed at improving health care for the poor often coincide with ethnic minority status. However, research on how health policies and reforms affect ethnic minorities based on ethnicity has been scarce. Toan et al. show that ethnic minorities use public health services to a lesser extent and attribute this to the failure of fee exemption and long distances to health services (35). There is also evidence for discrimination based on ethnicity within the health system (36), and there are few health workers from ethnic minority groups (37). Evidence for differential treatment of ethnic minorities is supported by findings even if ethnic minority mothers delivered at a health facility they were still subject to an increased risk of neonatal death (6). Furthermore, ethnic minorities received less expensive treatments and were less likely to undergo major surgery than Kinh patients, even though patients have the same disease, same age, and same gender.

It has also been suggested that there are financial barriers, such as informal fees, transportation costs, and expenditure on drugs, causing inequities between ethnic minorities and the majority Kinh (36). Wagstaff further points out that the HCFP does not remove many of the non-price constraints, such as geographical inaccessibility of facilities, that the targeted groups are faced with. At the same time, the program has a strong bias toward reimbursements for higher-level facilities and inpatient care, with a major part of the budget allocated to provincial and district hospitals (38). In addition, lower admission rates for inpatient care for ethnic minorities (39) contribute to maintaining inequity. The coverage of the program is also a source of inequity. Even if the program has three target groups: the poor, ethnic minorities living in targeted provinces, and households living in disadvantaged communes, Wagstaff shows that the program is mainly geared toward the poor, and unless they were poor, the two other groups were less covered. Overall the HCFP covered only 60% of those eligible in 2004 (38). There is also evidence that the implementation of governmental programs targeting ethnic minorities have been hampered by a lack of awareness about the existence of such programs among end-receivers (31).

Cultural perceptions about illness, language barriers, and discriminatory attitudes among health staff are factors to be considered as well, especially when it comes to care seeking and health care utilization. Teerawichitchainan and Phillips illustrated how ethnic minority parents were less likely to seek care when their children got sick, and that they were less likely to report severe illnesses in their children (40). Hong et al. came to similar conclusions when investigating the prevalence and treatment of childhood diarrhea, showing that being an ethnic minority mother was associated with a low level of health care utilization and that ethnic minority mothers were less likely to use oral rehydration solution (ORS) to treat children with diarrhea (41). In an effort to explain why health care utilization is poorer among ethnic minorities, Rheinlander et al. conducted a qualitative study in the northern province of Lao Cai. By interviewing 43 caregivers, and conducting three focus group discussions and participant observations at two commune health stations, they found a range of obstacles to seeking care facing ethnic minorities. Internal barriers, such as gender norms not allowing a woman to travel on her own, women lacking decision-making authority, or economic concerns about indirect costs and loss of income, influenced their health care utilization. However, external factors like long travelling distances and disrespectful and discriminatory behavior of health staff also constrained care-seeking. Nevertheless, the study did find that ethnic minority caregivers possessed the ability to accurately recognize danger signs of diarrhea, and that they simultaneously sought care from practitioners of traditional medicine (42). This picture was reinforced in a report from UNICEF analyzing the situation of children in rural province in northern Vietnam, where traditional health care beliefs and practices have been held up as an additional reason for ethnic minorities to be reluctant to use formal health care services (43).

Another attempt to gain a deeper understanding of the inequities in health care utilization is found in Oosterhoff et al. who investigated how guidelines and policies on reproductive health have affected the ethnic minority group, Black Thai, with a special focus on the cultural practice of temporary matrilocality (*zu kuay*). The Kinh practice of patrilocality and patrilinear norms has been promoted resulting in an elevated vulnerability for Thai women to marry HIV-positive drug users. The authors argue that the shortened period of *zu khay* results in women being more likely to unknowingly marry a man they did not know to be an intravenous drug user. The authors conclude that policies and guidelines must be adapted to ethnic minority traditions in order to be improved (44).

### Maternal, newborn and child health

Maternal and child health is an important focus of the Millennium Development Goals (MDGs) and a major public health concern. Ten articles and 10 documents from the grey literature were found evaluating ethnic minorities in this field (Table 3). The overall situation described in these articles is that ethnic minorities are generally worse off when it comes to maternal and child health. A WHO report from 2005 states that ethnic minority women face a four times higher risk of maternal mortality compared to Kinh women (45). The fertility rates are also higher among ethnic minorities, and women in these groups have been shown to use modern contraceptives to a lesser extent compared to Kinh women (26, 46). Two major contributors to higher fertility rates have been suggested: early childbearing and lower rates of abortion due to ideological objections (47). A qualitative study among H'mong minority women also found four major reasons for the lower rates of contraceptive use in this group: limited knowledge about contraceptives, fear of domestic violence, cultural taboos, and time constraints (48).

**Table 3:** Maternal, newborn and child health

Author(s)	Title	Publication/Agency	Year	Main results	Study design and sample	The ethnicity variable
Amin S, Teerawichitchainan B. (47)	Ethnic fertility differentials in Vietnam and their proximate determinants.	Working Paper No. 18, Population Council.	2009	Two major contributors to the higher fertility rate among ethnic minority groups are early childbearing and lower rates of abortion due to strong ideological opposition.	Vietnam National Health Survey (VNHS) 2001	Kinh-Chinese vs. Ethnic minority groups divided by region
Central population and housing census steering committee. (21)	The 2009 Vietnam Population and Housing census: Major findings.	Available at http://www.gso.gov.vn	2010	Infant mortality rate higher in ethnic minority groups.	Cross-sectional	Kinh, Tay, Thai, Muong, Khme, Mong and Other
Ekman B, Axelson H, Ha DA, Nguyen LT. (50)	Use of Maternal Health Care Services and Ethnicity: A Cross-Sectional Analysis of Vietnam.	Working papers series. Posted at SSRN June 15, 2007.	2007	The use of maternal health care services, including antenatal care, skilled assistance at birth and delivering at a clinic, is highly related to ethnicity such that ethnic minorities (EM) use significantly less of these services.	Cross-sectional using national survey data 2001/02	Kinh vs. Minority
Graner S, Klingberg-Allvin M, Phuc HD, Krantz G, Mogren I. (49)	The panorama and outcomes of pregnancies within a well-defined population in rural Vietnam 1999–2004.	Int J Behav Med. 2009: 16 (3):269–77.	2009	Higher risk of stillbirth for EM, OR 6.34 CI 1.33–30.29). EM more likely to delivery outside a health care facility (OR 1.85 adjusted for area of residence, economy, education and marital status)	Population-based surveys 1999, 2001, 2003. All women who reported a pregnancy from 1999 up to 2004 were included (n=5845)	Kinh vs. Minority
Hoa DP, Nga NT, Malqvist M, Persson LA. (7)	Persistent neonatal mortality despite improved under-five survival: a retrospective cohort study in northern Vietnam.	Acta Paediatr. 2008 Feb: 97 (2):166–70.	2008	Increased risk of neonatal mortality in ethnic minority groups, independent of economy and education. Increased risk o neonatal mortality over time.	Cross-sectional. Retrospective reproductive life stories were collected through interviews with 14329 women	Kinh vs. Minority
Knowles JC, Bales S, Cuong LQ, Oanh TTM, Luong DH. (59)	Health equity in Viet Nam: A situation analysis focused on maternal and child mortality.	UNICEF, Hanoi. Available at: http://www.un.org.vn/en/	2009	Child survival related to Vietnamese or Chinese ethnicity. Infant mortality even stronger associated to ethnicity.	Surveys of Population Change and Family Planning 2005 and 2006	Kinh/ Chinese vs. Minority
Ministry of Planning and Investment. (52)	Vietnam population and housing census 2009 – Sex ratio at birth in Vietnam: New evidence on patterns, trends and differentials	Available at: http://www.gso.gov.vn	2010	Data indicates that the SRB among minority (non-Kinh) women is relatively low at 105.9.	Cross-sectional survey	Kinh vs. Minority
Malqvist M, Nga NT, Eriksson L, Wallin L, Hoa DP, Persson LA. (6)	Ethnic inequity in neonatal survival: a case-referent study in northern Vietnam.	Acta Paediatr. 2011 Mar: 100 (3):340–6.	2011	Increased risk of neonatal mortality for ethnic minority mothers. The elevated risk was maintained even if the EM mothers went to hospital to deliver or if they attended ANC.	Case-referent study including 183 neonatal death cases and 599 referents.	Kinh vs. Minority
Malqvist M. (58)	Neonatal mortality: an invisible and marginalised trauma.	Glob Health Action. 2011 Mar 16: 4	2011	Increased risk of neonatal mortality for ethnic minority mothers, independent of distance, or place of delivery.	Case-referent study including 183 neonatal death cases and 599 referents.	Kinh vs. Minority
Malqvist M, Nga NT, Eriksson L, Wallin L, Ewald U, Persson LA. (53)	Delivery care utilisation and care-seeking in the neonatal period: a population-based study in Vietnam.	Ann Trop Paediatr. 2008 Sep: 28 (3):191–8.	2008	Ethnic minority mothers less likely to seek care at time of delivery.	Cross-sectional. Registry data. 284 neonatal death cases.	Kinh vs. Minority
Sepehri A, Sarma S, Simpson W, Moshiri S. (54)	How important are individual, household and commune characteristics in explaining utilization of maternal health services in Vietnam?	Soc Sci Med. 2008 Sep: 67 (6):1009–17.	2008	Kinh mothers are almost three times more likely to give birth at health facilities than EM women. While ethnicity has little influence on access to prenatal care it has a strong influence on a woman's decision on delivery location.	Cross-sectional using data from VNHS 2001, including 158 000 individuals in 36 000 households	Kinh vs. Minority
Teerawichitchainan B, Amin S. (46)	The role of abortion in the last stage of fertility decline in Vietnam.	Int Perspect Sex Reprod Health. 2010 Jun: 36 (2):80–9.	2010	EM in northern uplands and central highlands had higher fertility rates, were less likely to use modern contraceptives and had a lower abortion rate.	VNHS 2001 including 27097 women 15–49 years	Five clusters of ethnic groups; Kinh/Chinese, Tay/Thai/Muong/Nung (TTMN), EM in the south, EM in northern uplands, EM in central highlands.
UNFPA. (57)	Childbirth in ethnic minority communes – a qualitative study in Binh Dinh province.	Available at: http://www.vietnam.unfpa.org	2008	Poor understanding and knowledge about ethnic minority birth practices among health workers. Existing reproductive health services are not relevant to local customs and practices.	In-depth interviews, FGDs and non-participatory observations among health workers in Binh Dinh province	EM in Binh Dinh province.
UNFPA. (56)	Knowledge and behaviour of ethnic minorities on reproductive health	Available at: http://www.vietnam.unfpa.org	2007	Many ethnic minorities do not utilize delivery facilities due to a combination of complex rituals surrounding birth. Important not to attempt to explain complex situations with simple mono-causal factors.	Interviews and FGDs	EM in Ha Giang and Hoa Binh provinces
UNFPA. (48)	Reproductive health of H'mong people in ha Giang province.	Available at: http://www.vietnam.unfpa.org	2008	Low rates of contraceptive use due to limited knowledge, fear of domestic violence, cultural taboos and time constraints.	Ethnographic interviews, FGDs, observations and case studies	H'mong in Ha Giang province
UNICEF Vietnam and Dien Bien Provincial People's Committee. (43)	An analysis of the situation of children in Dien Bien.	Available at: http://www.un.org.vn/en/	2010	Ethnic minority mothers utilize healthcare services to a lesser extent. Explanations suggested are distance and cost, language barriers, the maintenance of traditional health care beliefs and a lack of knowledge.	FGDs and observational study	EM in Dien Bien Provence
UNICEF. (51)	An analysis of the situation of children in Viet Nam 2010.	Available at: http://www.un.org.vn/en/	2010	Low overall awareness of reproductive health among ethnic minority women.	Multiple Indicator Cluster Survey (MICS) 2 2006	Kinh vs. Minority
Vo Van T, Hoat LN, Jan van Schie T. (55)	Situation of the Kinh poor and minority women and their use of the Maternal Care and Family Planning Service in Nam Dong Mountainous District, Thuathien-Hue Province, Vietnam.	Rural Remote Health. 2004 Oct-Dec: 4 (4):255.	2004	Home delivery rate higher for EM (81%) compared to Kinh (19%). Illiteracy rate higher among EM. Language was a common barrier when Kinh health staff attempted to communicate with minority women. Most communication material at the health centre was written in Kinh.	Oral questionnaire. Random sample of 420 women with children under 5 in seven communes	Kinh vs. Minority
World Health Organization (WHO). (26)	Health and ethnic minorities in Viet Nam.	Technical Series No. 1	2003	Most provinces with high infant and child mortality rates have high concentrations of EM.	Population Census 1999	Comparing 10 provinces with highest and lowest percentage of EM
World Health Organization (WHO). (45)	Maternal mortality in Viet Nam 2000–2001: An in-depth analysis of causes and determinants.	Available at: http://dosei.who.int/	2005	Women in ethnic minority groups had a relative risk of maternal death 3.92 times higher than Kinh women.	Cross-sectional survey in seven provinces	Kinh vs. Minority

There is also evidence of higher stillbirth rates (49) and less use of antenatal care services among ethnic minority groups (50, 51). However, sex ratio at birth is not significantly elevated among ethnic minorities, which has instead been the case in the general population in recent years (52). The place of delivery is important in relation to health and survival of both mother and child, and a number of studies show that ethnic minority mothers are more likely to deliver at home (49, 50, 53–55). There are a variety of explanations for the preference for home delivery in ethnic minority groups, such as distance and cost of going to the health center, informal payments for treatment and unfamiliarity with the procedures in combination with language barriers, and negative attitudes among health staff (43). However, other studies claim that many of these explanations are rationalizations, since distance is relative, and that the ‘shyness’ of ethnic minority women that is often referred to is actually rooted in other factors (56). Complex rituals surrounding child birth and strong traditional beliefs in combination with patriarchic structures are instead presented as the main reason for low facility delivery rates among ethnic minority groups (43, 56). It has also been pointed out that there is a low level of knowledge and understanding about these culturally specific practices among health staff, exacerbating difficulties in adapting practice (57).

Since home deliveries are associated with an increased risk of neonatal mortality, it is expected that neonatal mortality rates will be higher in ethnic minority groups. Hoa et al. and Malqvist et al. verified the increased risk of neonatal death in the ethnic minority groups compared to the Kinh group, independent of economic status and maternal education (6, 7, 58). Furthermore, Malqvist et al. also showed that even if ethnic minority mothers delivered at a health facility, the increased risk was maintained (6) irrespective of distance to health facility (58). Language barriers and differences in cultural traditions and perceptions have been offered as explanations to discrepancies in quality of care received (55, 58). For example, there is no health information material at the local health stations written in any minority language.

In an extensive report based on secondary analyses from existing data, Knowles et al. investigated health equity in Vietnam with a focus on maternal and child health. By constructing concentration curves and indices, they showed that child survival was moderately associated with ethnic minority status. Infant mortality on the contrary, showed a strong association with minority status (59).

### Nutrition

In the field of nutrition, seven publications meeting the inclusion criteria were found, mostly dealing with child nutrition, measuring stunting and wasting (Table 4). Three studies found an association between ethnic minority status and child malnutrition, but none of the studies had performed appropriate regression or stratification to find possible confounders (60, 61). One of the papers suggested that geographical location is likely to influence the prevalence of stunting (61). This was verified by Thang and Popkin who showed that living in a rural area more than doubled the risk of a child being stunted (62). The authors also demonstrated in another article that minority populations consume less calories and eat less food rich in starches, lipids, and proteins (63), but that household economy is more strongly associated to stunting than ethnicity (64).

**Table 4:** Nutrition

Author(s)	Title	Publication	Year	Main results	Study design and sample	The ethnicity variable
Bui QT, Le Linh C, Rahman Z. (60)	Child health status and maternal and child care in Quangtri Province, Vietnam.	Asia Pac J Public Health. 2008 Oct: 20 Suppl:228–35.	2008	Ethnicity associated with child malnutrition	Cross-sectional, FGDs and In-depth interviews. 400 mothers with children under 2. 27 mothers interviewed and 4 FGDs with total 38 participants	Kinh vs. Minority
Haughton D, Haughton J. (61)	Explaining Child Nutrition in Vietnam.	Economic Development and Cultural Change, Vol. 45, No. 3 (April 1997), pp. 541–556.	1997	Ethnic minorities (EM) more likely to be stunted (-0,2 SD), possibly due to geography	Vietnam Living Standard Survey (VLSS) 1992–93. 7046 children from 4800 households	Kinh vs. Minority
Thang NM, Popkin B. (64)	Child malnutrition in Vietnam and its transition in an era of economic growth.	J Hum Nutr Diet. 2003 Aug: 16 (4):233–44.	2003	Minority populations living in rural areas were more likely to be stunted. Minority status not predictive for escaping stunting. Economy showing stronger association	Cross-sectional using data from VLSS 1992–93 and 1997–98. 4305 households and 4367 children	Kinh vs. Minority
Thang NM, Popkin BM. (63)	Patterns of food consumption in Vietnam: effects on socioeconomic groups during an era of economic growth.	Eur J Clin Nutr. 2004 Jan: 58 (1):145–53.	2004	EM consumes 19.3 kcal/day less than Kinh population. EM populations not only eat less foods rich in proteins and lipids, but also less food rich in starches	Cross-sectional using household from VLSS 1992–93 and individuals from VLSS 1997–98 23839 individuals (1992–93) and 28509 individuals (1997–98)	Kinh vs. Minority
Thang NM, Popkin BM. (62)	In an era of economic growth, is inequity holding back reductions in child malnutrition in Vietnam?	Asia Pac J Clin Nutr. 2003: 12 (4):405–10.	2003	EM living in rural areas were 2.34 times more likely to be stunted. No difference only from minority status	Cross-sectional using data from VLSS 1997–98. 5309 children	Kinh vs. Minority
Trinh LT, Dibley M. (65)	Anaemia in pregnant, postpartum and non-pregnant women in Lak district, Daklak province of Vietnam.	Asia Pac J Clin Nutr. 2007: 16 (2):310–5.	2007	Women of BoY, Ede and Koho minorities had an increased risk of anaemia compared to Kinh women (OR 2.7 CI 1.4–5.0). No difference between Kinh and M'nong overall, but pregnant M'nong women more likely to be anemic than Kinh (adjOR 2.1 CI 1.2–9.2)	Cross-sectional 2001 in a mountainous area. All pregnant and post pregnant women (up to 6 months after delivery) were included. An equal amount of non-pregnant women	M'nong (62%), Kinh (31%), BoY, Ede and Koho (7%)
UNICEF. (51)	An analysis of the situation of children in Viet Nam 2010.	Available at: http://www.un.org.vn/en/	2010	Underweight malnutrition is considerably higher among ethnic minority children compared to Kinh (30 per cent versus 18 per cent)	Multiple Indicator Cluster Survey (MICS) 2 2006	Kinh vs. Minority

Trinh and Dibley investigated anaemia in pregnant, postpartum, and non-pregnant women in a mountainous province in the central highlands and found that ethnic minority status was associated with anemia in three of the four ethnic minority groups included (65).

### Infectious diseases

Eight publications dealt with infectious diseases and were included for this area (Table 5), with three sub-themes: HIV/TB (5), Malaria (1), and Neglected diseases (2).

**Table 5:** Infectious diseases

Author(s)	Title	Publication	Year	Main results	Study design and sample	The ethnicity variable	Sub theme
Abe T, Honda S, Nakazawa S, Tuong TD, Thieu NQ, Hung le X, Thuan le K, Moji K, Takagi M, Yamamoto T. (71)	Risk factors for malaria infection among ethnic minorities in Binh Phuoc, Vietnam.	Southeast Asian J Trop Med Public Health. 2009 Jan: 40 (1):18-29.	2009	Ethnic minority status not associated with malaria infection.	Cross-sectional study including 682 individuals in a village in Binh Phuoc province in southern Vietnam.	Kinh (20%) vs. Stieng (80%)	Malaria
Bui TD, Pham CK, Pham TH, Hoang LT, Nguyen TV, Vu TQ, Detels R. (69)	Cross-sectional study of sexual behaviour and knowledge about HIV among urban, rural and minority residents in Viet Nam.	Bulletin of the World Health Organization, 2001; 79 (1):15–21.	2001	Lower awareness about HIV in areas with a large proportion of ethnic minorities.	Cross-sectional	Kinh vs. Minority	HIV/TB
Des Jarlais DC, Johnston P, Friedmann P, Kling R, Liu W, Ngu D, Chen Y, Hoang TV, Donghua M, Van LK, Tung ND, Binh KT, Hammett TM. (68)	Patterns of HIV prevalence among injecting drug users in the cross-border area of Lang Son Province, Vietnam, and Ning Ming County, Guangxi Province, China.	BMC Public Health. 2005 Aug 24: 5:89.	2005	Ethnicity was not related to HIV status.	Cross-sectional survey of injecting drug users in Lan Son province in northern Vietnam. (n=348)	Kinh vs. Minority	HIV/TB
Hammett TM, Johnston P, Kling R, Liu W, Ngu D, Tung ND, Binh KT, Dong HV, Hoang TV, Van LK, Donghua M, Chen Y, Des Jarlais DC. (67)	Correlates of HIV status among injection drug users in a border region of southern China and northern Vietnam.	J Acquir Immune Defic Syndr.	2005	Ethnicity was not related to HIV status.	Cross-sectional survey of injecting drug users in Lan Son province in northern Vietnam. (n=348)	Kinh vs. Minority	HIV/TB
Huong NT, Vree M, Duong BD, Khanh VT, Loan VT, Co NV, Borgdorff MW, Cobelens FG. (15)	Delays in the diagnosis and treatment of tuberculosis patients in Vietnam: a cross-sectional study.	BMC Public Health. 2007 Jun 13: 7:110.	2007	Patients’ delay longer in ethnic minority groups.	Cross-sectional survey of new TB patients at 70 randomly selected districts. (n=2093)	Kinh vs. Minority	HIV/TB
Schratz A, Pineda MF, Reforma LG, Fox NM, Le Anh T, Tommaso Cavalli-Sforza L, Henderson MK, Mendoza R, Utzinger J, Ehrenberg JP, Tee AS. (73)	Neglected diseases and ethnic minorities in the Western Pacific Region exploring the links.	Adv Parasitol. 2010: 72:79–107.	2010	Coincidental report of high prevalence of soil transmitted helminth infections in areas with large ethnic minority populations.	Epidemiological data.	Kinh vs. Minority	Neglected diseases
Vach TH, Cuong ND. (66)	Study on access to care, treatment, and support for children and women with HIV and AIDS among communities with higher numbers of ethnic minority people in Dien Bien, Kon Tum, and An Giang provinces.	UNICEF, Research Centre for Rural Population and Health and Ministry of Health; available at www.vaac.gov.vn	2010	Equitable and universal access among vulnerable groups, including ethnic minority children and women related to HIV/AIDS control and prevention has not been well addressed.	In-depth interviews and Focus Groups Discussions (FGDs).	Ethnic minorities (EM) in Dien Bien, Kon Tum and An Giang provinces	HIV/TB
Verle P, Kongs A, De NV, Thieu NQ, Depraetere K, Kim HTM, Dorny P. (74)	Prevalence of intestinal parasitic infections in northern Vietnam.	Tropical Medicine & International Health 8(10) 1365–3156.	2003	Prevalence of nematode infections was high in all ethnic groups.	Cross-sectional study including 2686 individuals from 50 villages in the province of Hoa Binh.	Muong (64%), Kinh (11%), Dao (10%), Thai (6%), Tay (5%), Hmong (4%)	Neglected diseases

There is an extensive volume of research performed in the area of HIV/AIDS and TB in Vietnam. However, the material dealing with distribution and vulnerability of HIV and TB in relation to ethnic minorities is scarce (66). Only five publications were found that met the inclusion criteria (Table 5). Des Jarlais et al. and Hammet et al. use the same material analysing cross-sectional data from a border province in northern Vietnam. They found no association between ethnic minority status and HIV status (67, 68). Bui et al. also used cross-sectional interviews with 630 people from 707 households in Quang Ninh province, also bordering China in the north. They found a low awareness about HIV in areas with a large proportion of ethnic minorities, even if the link to minority status was not clear (66, 69). A UNICEF report also states that even if there have been strong policies by the Vietnamese government to provide HIV and AIDS treatment and care for people living with HIV, and equitable access among vulnerable groups like ethnic minorities, these have not yet been implemented (66).

Malaria was highly endemic in Vietnam in the early 1990s, but a national program for malaria control launched in 1992 managed to reduce mortality and morbidity with activities, such as early diagnosis and treatment, bed net distribution, and indoor-residual spraying (70). Nonetheless, malaria is still a health hazard, especially in the remote and mountainous areas in the south inhabited by ethnic minorities. Abe et al. did not find an association between malaria and ethnic minority status, (71) even if there are examples of the low perception of malaria risk among certain ethnic minority groups, as illustrated by Peeters et al. (72).

The prevalence of neglected tropical diseases, mainly parasitic infections, has been shown to be high in areas with a high proportion of ethnic minorities (73). However, the evidence is coincidental and the findings could most likely be explained by living standard and economy. For example, Verle et al. did not find any differences between ethnic groups when investigating the prevalence of intestinal parasitic infections in northern Vietnam (74).

### Oral health and hygiene

Three publications that met the inclusion criteria dealt with oral health and hygiene (Table 6). Uetani et al. have investigated the oral health status of vulnerable groups in the central highlands of southern Vietnam and compared the Kinh majority and a group of Co-Ho minority people. Their results indicate that dental health status was low in both groups, with a high prevalence of caries, and a tendency for the Kinh group below the age of 30 to be somewhat worse off. Their conclusions and recommendations indicate the need to improve oral health among rural populations (75).

**Table 6:** Oral health and hygiene

Author(s)	Title	Publication	Year	Main results	Study design and sample	The ethnicity variable
Rheinländer T, Samuelsen H, Dalsgaard A, Konradsen F. (78)	Hygiene and sanitation among ethnic minorities in Northern Vietnam: does government promotion match community priorities?	Soc Sci Med. 2010 Sep: 71 (5):994–1001.	2010	Cultural perceptions of hygiene and sanitation did not differ substantially and were similar to hygiene explanations found in the rural majority population elsewhere in Vietnam. However, in order to design effective sanitation programs, policy makers and program managers need to understand the underlying cultural and social factors that determine sanitation behaviour and priorities, for example, by allowing for a larger diversity of low-cost sanitation solutions.	Participatory observations in 4 villages and 20 case households over a period of six months (May-October 2008). In addition, 10 key informants and 60 household-members were interviewed and 4 focus group discussions conducted	Representatives from Giáy, Tày and Xá Phó (all lowland villages), and red Dao.
Uetani M, Jimba M, Kaku T, Ota K, Wakai S. (75)	Oral health status of vulnerable groups in a village of the Central Highlands, southern Vietnam.	Int J Dent Hyg. 2006 May: 4 (2):72–6.	2006	The Co-Ho were less likely to have heard about oral health than the Kinh (OR: 6.0; 95%CI: 1.2–14; P=0.014). The younger group of the Kinh had more dental caries than the Co-Ho (OR: 1.8; 95%CI: 1.5–2.2; P=0.001).	195 participants from 56 households	Kinh vs. Co-Ho
UNICEF and Viet Nam Administration of Preventive Medicine. (76)	A summary of national baseline survey on environmental sanitation and hygiene situation in Viet Nam.	Available at: http://www.un.org.vn/en/	2007	The probability of ethnic minority households to have hygienic latrines is 12 folds lower than for Kinh households.	Cross-sectional survey of 37 306 households	Kinh vs. Minority

Sanitation and hygiene are important in the efforts to prevent infectious diseases. To increase affordability of proper sanitation, the Ministry of Health in Vietnam subsidizes the construction of standardized models of latrines. Hygiene promotion has been added to this government driven subsidy program, mainly in the form of information material and training of village health workers to become hygiene advocators. Ethnic minorities have less access to hygienic latrines, and it is imperative to improve the standard within these groups (76).

To be effective, health and hygiene initiatives need to be culturally and socially appropriate (77). Therefore, Rheinländer et al. investigated how government promotion activities have been received along with cultural appropriateness in four ethnic minority villages. They concluded that the perceptions of hygiene and sanitation guiding the daily practices in these groups did not deviate from what could be found in other rural populations, including Kinh groups. Thus, the recommended latrines are met with reluctance due to perceptions of the body as permeable and vulnerable to bad smells and ‘dirty air’. The authors also concluded that the present government program pacifies the ethnic minority groups by reinforcing the notion that hygiene ‘comes from the outside’, and they recommended that future hygiene interventions are tailored to better fit community priorities (78).

## Discussion and conclusions

This review points at a severe inequity in health along ethnic lines in Vietnam. The explanations offered to this situation are derived from both external and internal sources. Governmental policies and programs have not been culturally adapted and sensitive, and there are reports of bad attitudes and discrimination from health staff toward minority people. At the same time, there are examples of traditions and patriarchic structures within ethnic minority groups that also maintain harmful health behaviors.

We believe that we have included all relevant resources. However, in the literature, the distinction between circumstantial evidence, reporting of previously reported findings and relevant accounts of ethnic minority health issues have sometimes not been clear, which might have affected the selection process. Many of the studies presented lacked a data analysis strategy to distinguish the effects of ethnicity from the influence of other important socioeconomic factors, for example, by adequately adjusted regression models or stratification schemes. These studies do however contribute to the aim of this review by adding to the overall picture of ethnic minority health in Vietnam. The diverse research interests and areas of the studies included did not allow for meta-analysis or any other statistical compilation. Therefore, the results are presented as a narrative review although the search methods and criteria were set up as a systematic review (34). This review has only included articles and documents presented in English. Doctoral or master theses are not included. This is of course a limitation to consider, but logistic constraints related to translation have hampered this effort.

Some topics lack an ethnic analysis more than others, such as the relation of maternal mortality and other obstetric outcomes. HIV/AIDS and TB are also areas with surprisingly low levels of ethnic analysis (66). Only four articles on HIV/AIDS and one on TB could be identified that met the inclusion criteria. In these already well researched areas we would like to encourage a more holistic and cross-cutting approach, taking socioeconomic determinants and policy issues into account. Currently, the focus on ethnic inequity and health is to a large extent dependent on researchers’ interests, as illustrated by the fact that three out of seven articles included under nutrition were from the same author. One area of major public health interest that is lacking completely is mental health. Even descriptive studies on the mental health situation are scarce in Vietnam, even more so studies addressing the issue in disadvantaged populations (79).

However, even if there is still a lack of quantitative studies to distinguish between the effects of ethnicity on health from that of poverty and lack of education, it is non-controversial to claim that ethnicity plays an important role as a determinant of health. What is therefore needed is more research on the reasons and mechanisms of this influence (80). Qualitative research is best suited to answer the questions ‘why’ and ‘how’, but only a few qualitative studies addressing this issue in relation to health were found. To design interventions and take affirmative action, this knowledge is needed.

A common theme and re-emerging issue on the reviewed literature was policy development and implementation. An extensive summary of the development from *doi moi* up until the turn of the century demonstrates how the otherwise relatively successful development strategies were less successful in the areas of social services and health care (9). Policies of exemption, where poor families should be guaranteed health care free of charge, were not applied effectively. For example, the health insurance system up until 2003 did not function as intended since only a minor part (6%) of the poorest quintile were insured at the time (81). In 2003, the Health Care Fund for the Poor (HCFP) was launched in response to this situation. A selected sample of disadvantaged communes, mostly in the remote mountainous areas with high proportion ethnic minorities was targeted. About 15% of the total population was eligible for the benefits of HCFP by this approach, and the impact has been ambiguous. Initial analyses indicated that there were positive changes in health care utilization and a reduced risk of catastrophic spending (16, 82) in the prioritized communes. However, out-of-pocket expenditure was increased in the short term after the introduction of HCFP (82). More recent results have not found any evidence for changes in the use of services, instead they found a substantial reduction of out-of-pocket expenditure (38). Regardless of the outcome of the HCFP, it is a good illustration of how ethnic diversity has been neglected, by imposing the same scheme in all the selected areas, which might be a reason for its ambiguous impact.

Other authors reviewed in this paper have testified about policies that disregard local traditions and customs in such diverse areas as reproductive health (44), malaria (72), and sanitation (42). This may be an expression of the hegemonic perception of ethnic minorities among the Kinh as described by ethnographers (33). In combination with a socialist ideology emphasizing Vietnamese unity and promoting the Kinh tradition and culture as norm, these perceptions have, according to McElwee, generated policies and programs intentionally directed toward ethnic minorities that in the end manifest the majority culture (30). Thus, the efforts from health care planners and policy makers, while well intentioned, have likely reinforced the horizontal ethnic inequity in health.

In general, we found a small number of studies that considered ethnicity. Most studies that addressed equity used different measures of economic status as the main independent variable; even if data on ethnicity were collected, such as in the Demographic and Health Surveys of 2002, they were seldom analyzed. For example, in the baseline for monitoring the effects of program 135, data were collected for 11 different ethnic groups. However, although the author pointed out that it would be a unique opportunity to disaggregate ethnic minorities (p. 17), all minority groups in the analysis were still grouped together as one. The authors of the final report instead suggested that an analysis based on different ethnic minority groups could be subject to future research (83). With this in mind, more attention to ethnicity as one of the major socioeconomic determinants is needed to get a more valid and comprehensive picture of ethnic minority health in Vietnam (80, 84).

In our review, we also found a tendency to analyze data according to geographical division and to equalize areas with high density of ethnic minorities with ethnicity itself. This was particularly predominant in government documents and other grey literature. By doing so, the effects of ethnicity will be reduced to a set of proximate determinants restricted to geographical area, which will underestimate or hide the horizontal dimension of ethnicity. The targeting of certain areas with large proportions of minority people rather than minority groups suggests that efforts to improve ethnic minority health are driven by an assimilation policy rather than cultural specific strategies (32). This is further illustrated by the fact that many services and related information are not made available in the local language. Historically, Vietnamese culture has been favored and there are examples of assimilation policies promoting Vietnamese culture and Vietnamese language (29, 85). This is an aspect that needs to be taken into account when performing research on ethnic minority issues in Vietnam. Vietnam, as other socialist countries, has a strong hierarchical culture, and even if society in recent years has opened up, there is still evidence that bureaucracy and governmental policy puts constraints on research in minority areas (86, 87).

It is not only in policy formulation that the ethnic dimension is reduced. There is a strong tendency in the reviewed literature to group ethnic minorities together and refer to them as just ‘minority’, without any critical reflection over the inherent discrepancies between different ethnic minority groups (see ‘ethnicity variable’ in tables). This grouping of ethnic minorities most likely has its roots in the common perception among the Kinh majority of ethnic minorities being essentially one entity. Minorities are portrayed both in the official school system and in modern media as essentially different from Kinh culture (33). This notion of Otherness group even widely disparate cultures together to a perceived homogeneity with a common denominator of being poor, underdeveloped, and helpless, and at the same time colorful and exotic (33). Only seven of the quantitative studies reviewed had a different division than Kinh/Minority. In two studies, the Chinese minority was grouped together with Kinh due to its higher socioeconomic status (40, 59), and five studies took on a more detailed approach defining the ethnic groups and/or trying to divide the different ethnic group in their analysis (46, 65, 71, 74, 75). Not to disaggregate data by ethnicity will promote invisibility of ethnic inequity and contribute to the structural violence it is expressing.

Many of the ethnic minorities in Vietnam can be considered indigenous and suffer from the same mechanisms of inequality as other indigenous people around the globe. The combination of classic socioeconomic deficits and culturally and historically specific factors that give rise to indigenous health disparities (88) holds true also for the ethnic minority groups of Vietnam. Most ethnic minority groups are poorer and less educated and thereby affected by health inequalities due to lower socioeconomic status. But ethnic status affects the health of these groups over and above economic status. In general, ethnicity, as a marker of group belonging, has some benefits to its members, such as social stability and a sense of belonging (89). However, in the social hierarchies where ethnicity has a role, marginalized ethnic groups are also trapped and subject to oppressive structures (29). Stewart labels this as horizontal inequality; the fact that inequality exist on the basis of a group rather than between individuals (vertical inequality) (90). The horizontal inequality, or the social structure, is maintained by both internal and external factors. Internally, traditions and norms within a group that strengthen group belonging may affect health-seeking behavior or the introduction of evidence-based medicine as described above (42). Externally, the group can be subject to discrimination and adverse attitudes from the hegemonic outsiders, resulting in lower quality of care and inferior access to health services (36). When these horizontal inequalities result in differing health outcomes, it becomes a case of horizontal inequity, since the difference in health outcome is derived from social position and is therefore unjust (91). This group dimension, and the resulting horizontal inequity, has played a minor role in efforts to improve public health. The MDGs are individual focused and concerned primarily with numbers of individuals reached or saved. This diverts the focus of public health efforts to a utilitarian approach where improvements are attempted within existing societal structures, rather than intending to alter conserving structures. The example of horizontal inequity in this study, the health of ethnic minorities in Vietnam, highlights the need for affirmative action as a part of public health efforts in order to truly target inequity in health (92).

To overcome the ethnic inequities in health, not only in Vietnam, there is a need to better understand the scope and pathways of horizontal inequity. Awareness of ethnicity in itself as a determinant of health, not only as a covariate to poverty or living area, needs to be raised, and research needs to be designed with this in mind.

**Key messages**

- Ethnicity needs to be highlighted as a determinant of inequity in health, over and above income and education.
- An emphasis on horizontal, as opposed to vertical, inequities might be a way to display hitherto neglected aspects of minority health.
- There is a severe lack of information and research to understand how these inequities are mediated and affect each ethnic group.
- Without such research agenda, public policies in the sector of health will continue to target ethnic minorities inefficiently.

## References

[1] Gao J, Qian J, Tang S, Eriksson BO, Blas E (2002). Health equity in transition from planned to market economy in China. Health Policy Plan.

[2] Liu Y, Hsiao WC, Eggleston K (1999). Equity in health and health care: the Chinese experience. Soc Sci Med.

[3] World Health Organization (2011). Closing the gap: policy into practice on social determinants of health: discussion paper. World Conference on Social Determinants of Health; 19–21 October 2011.

[4] Granlund D, Chuc NT, Phuc HD, Lindholm L (2010). Inequality in mortality in Vietnam during a period of rapid transition. Soc Sci Med.

[5] Marmot M, Friel S, Bell R, Houweling TA, Taylor S (2008). Closing the gap in a generation: health equity through action on the social determinants of health. Lancet.

[6] Malqvist M, Nga NT, Eriksson L, Wallin L, Hoa DP, Persson LA (2011). Ethnic inequity in neonatal survival: a case-referent study in northern Vietnam. Acta Paediatr.

[7] Hoa DP, Nga NT, Malqvist M, Persson LA (2008). Persistent neonatal mortality despite improved under-five survival: a retrospective cohort study in northern Vietnam. Acta Paediatr.

[8] Thorson A, Hoa NP, Long NH (2000). Health-seeking behaviour of individuals with a cough of more than 3 weeks. Lancet.

[9] Gabriele A (2006). Social services policies in a developing market economy oriented towards socialism: the case of health system reforms in Vietnam. Rev Int Polit Econ.

[10] Hoang MV, Nguyen TB, Kim BG, Dao LH, Nguyen TH, Wright P (2008). Cost of providing the expanded programme on immunization: findings from a facility-based study in Viet Nam, 2005. Bull World Health Organ.

[11] Murakami H, Van Cuong N, Van Tuan H, Tsukamoto K, Hien do S (2008). Epidemiological impact of a nationwide measles immunization campaign in Viet Nam: a critical review. Bull World Health Organ.

[12] Huong DB, Phuong NK, Bales S, Jiaying C, Lucas H, Segall M (2007). Rural health care in Vietnam and China: conflict between market reforms and social need. Int J Health Serv.

[13] Thuan NT, Lofgren C, Lindholm L, Chuc NT (2008). Choice of healthcare provider following reform in Vietnam. BMC Health Serv Res.

[14] Tuan T, Dung VT, Neu I, Dibley MJ (2005). Comparative quality of private and public health services in rural Vietnam. Health Policy Plan.

[15] Huong NT, Vree M, Duong BD, Khanh VT, Loan VT, Co NV (2007). Delays in the diagnosis and treatment of tuberculosis patients in Vietnam: a cross-sectional study. BMC Public Health.

[16] Wagstaff A (2007). Health insurance for the poor: initial impacts of Vietnam's Health Care Fund for the Poor. World Bank Working Paper Series 4143, Impact Evaluation Series No. 11.

[17] Ekman B, Liem NT, Duc HA, Axelson H (2008). Health insurance reform in Vietnam: a review of recent developments and future challenges. Health Policy Plan.

[18] Chongsuvivatwong V, Phua KH, Yap MT, Pocock NS, Hashim JH, Chhem R (2011). Health and health-care systems in southeast Asia: diversity and transitions. Lancet.

[19] Committee for Population Family and Children [Vietnam], and ORC Macro (2003). Vietnam demographic and health survey 2002.

[20] General Statistics Office, National Institute of Hygiene and Epidemiology (NIHE) [Vietnam], and ORC Macro (2006). Vietnam population and AIDS indicator survey 2005.

[21] Central Population and Housing Census Steering Committee (2010). The 2009 Vietnam population and housing census: major findings.

[22] General Statistics Office (2006). Result of the Viet Nam household living standards survey 2006.

[23] General Statistics Office (2008). Result of the survey on household living standards 2008.

[24] Malqvist M, Eriksson L, Nga NT, Fagerland LI, Hoa DP, Wallin L (2008). Unreported births and deaths, a severe obstacle for improved neonatal survival in low-income countries; a population based study. BMC Int Health Hum Rights.

[25] Huy TQ, Long NH, Hoa DP, Byass P, Ericksson B (2003). Validity and completeness of death reporting and registration in a rural district of Vietnam. Scand J Public Health Suppl.

[26] World Health Organization (2003). Health and ethnic minorities in Viet Nam.

[27] Baulch B, Chuyen T, Haughton D, Haughton J (2007). Ethnic minority development in Vietnam. J Dev Stud.

[28] Imai K, Gaiha R (2007). Poverty, inequalities and ethnic minorities in Vietnam.

[29] Taylor P (2008). Minorities at large; new approaches to minority ethnicity in Vietnam.

[30] McElwee P, Taylor P (2008). “Blood relatives” or uneasy neighbours? Kinh migrant and ethnic minority interactions in the Truong Son Mountains. Minorities at large; new approaches to minority ethnicity in Vietnam.

[31] Committee of Ethnic Minorities, UNICEF (2003). Reaching out for change: a qualitative assessment of government health care and education policies affecting the women and children of ethnic minorities.

[32] Volkmann CS (2006). Children's rights and the MDGs: the right to health within Vietnam's transition towards a market economy. Health Hum Rights.

[33] Hanh DB, Taylor P (2008). Contesting marginality: consumption, networks, and everyday practice among Hmong Girls in Sa Pa, Northwestern Vietnam. Minorities at large; new approaches to minority ethnicity in Vietnam.

[34] Collins JA, Fauser BC (2005). Balancing the strengths of systematic and narrative reviews. Hum Reprod Update.

[35] Toan NV, Trong LN, Hojer B, Persson LA (2002). Public health services use in a mountainous area, Vietnam: implications for health policy. Scand J Publ Health.

[36] Castel P (2009). Vietnam Health Insurance: use of health care services by the poor efficiency and equity issues in the province of Kon Tum. SSRN eLibrary.

[37] Ministry of Health, Health Partnership Group (2009). Joint annual health review 2009 – human resources for health in Vietnam.

[38] Wagstaff A (2010). Estimating health insurance impacts under unobserved heterogeneity: the case of Vietnam's health care fund for the poor. Health Econ.

[39] Ministry of Health, Health Partnership Group (2010). Joint annual health review 2010 – Vietnam's health system on the threshold of the five-year plan 2011–2015.

[40] Teerawichitchainan B, Phillips JF (2008). Ethnic differentials in parental health seeking for childhood illness in Vietnam. Soc Sci Med.

[41] Hong TK, Dibley MJ, Tuan T (2003). Factors affecting utilization of health care services by mothers of children ill with diarrhea in rural Vietnam. Southeast Asian J Trop Med Public Health.

[42] Rheinlander T, Samuelsen H, Dalsgaard A, Konradsen F (2011). Perspectives on child diarrhoea management and health service use among ethnic minority caregivers in Vietnam. BMC Public Health.

[43] UNICEF Dien Bien Provincial People's Committee (2010). An analysis of the situation of children in Dien Bien.

[44] Oosterhoff P, White J, Thi Huong N (2011). Family health consequences of modernisation programmes in Black Thai communities. Cult Health Sex.

[45] World Health Organization (2005). Maternal mortality in Viet Nam 2000–2001: an in-depth analysis of causes and determinants.

[46] Teerawichitchainan B, Amin S (2010). The role of abortion in the last stage of fertility decline in Vietnam. Int Perspect Sex Reprod Health.

[47] Amin S, Teerawichitchainan B (2009). Ethnic fertility differentials in Vietnam and their proximate determinants. Working Paper No. 18.

[48] UNFPA (2008). Reproductive health of H'mong people in Ha Giang province – medical anthropology perspective.

[49] Graner S, Klingberg-Allvin M, Phuc HD, Krantz G, Mogren I (2009). The panorama and outcomes of pregnancies within a well-defined population in rural Vietnam 1999–2004. Int J Behav Med.

[50] Ekman B, Axelson H, Ha D, Liem N (2007). Use of maternal health care services and ethnicity: a cross-sectional analysis of Vietnam. SSRN eLibrary.

[51] UNICEF (2010). An analysis of the situation of children in Viet Nam 2010.

[52] Ministry of Planning and Investment (2010). Vietnam population and housing census 2009 – sex ratio at birth in Vietnam: new evidence on patterns, trends and differentials.

[53] Malqvist M, Nga NT, Eriksson L, Wallin L, Ewald U, Persson LA (2008). Delivery care utilisation and care-seeking in the neonatal period: a population-based study in Vietnam. Ann Trop Paediatr.

[54] Sepehri A, Sarma S, Simpson W, Moshiri S (2008). How important are individual, household and commune characteristics in explaining utilization of maternal health services in Vietnam?. Soc Sci Med.

[55] Vo Van T, Hoat LN, Jan van Schie T (2004). Situation of the Kinh poor and minority women and their use of the Maternal Care and Family Planning Service in Nam Dong Mountainous District, Thuathien-Hue Province, Vietnam. Rural Remote Health.

[56] UNFPA (2007). Knowledge and behaviour of ethnic minorities on reproductive health.

[57] UNFPA (2008). Childbirth in ethnic minority communities – a qualitative study in Binh Dinh province.

[58] Malqvist M (2011). Neonatal mortality: an invisible and marginalised trauma. Glob Health Action.

[59] Knowles JC, Bales S, Cuong LQ, Oanh TTM, Luon DH Health Equity in Viet Nam: a situation analysis focused on maternal and child mortality. Background paper prepared for the UNICEF Consultancy on Equity in Access to Quality Healthcare for Women and Children, April 8–10, 2009.

[60] Bui QT, Le Linh C, Rahman Z (2008). Child health status and maternal and child care in Quangtri Province, Vietnam. Asia Pac J Public Health.

[61] Haughton D, Haughton J (1997). Explaining child nutrition in Vietnam. Econ Dev Cult Change.

[62] Thang NM, Popkin BM (2003). In an era of economic growth, is inequity holding back reductions in child malnutrition in Vietnam?. Asia Pac J Clin Nutr.

[63] Thang NM, Popkin BM (2004). Patterns of food consumption in Vietnam: effects on socioeconomic groups during an era of economic growth. Eur J Clin Nutr.

[64] Thang NM, Popkin B (2003). Child malnutrition in Vietnam and its transition in an era of economic growth. J Hum Nutr Diet.

[65] Trinh LT, Dibley M (2007). Anaemia in pregnant, postpartum and non pregnant women in Lak district, Daklak province of Vietnam. Asia Pac J Clin Nutr.

[66] Vach TH, Cuong LQ (2010). Study on access to care, treatment, and support for children and women with HIV and AIDS among communities with higher numbers of ethnic minority people in Dien Bien, Kon Tum, and An Giansg provinces.

[67] Hammett TM, Johnston P, Kling R, Liu W, Ngu D, Tung ND (2005). Correlates of HIV status among injection drug users in a border region of southern China and northern Vietnam. J Acquir Immune Defic Syndr.

[68] Des Jarlais DC, Johnston P, Friedmann P, Kling R, Liu W, Ngu D (2005). Patterns of HIV prevalence among injecting drug users in the cross-border area of Lang Son Province, Vietnam, and Ning Ming County, Guangxi Province, China. BMC Public Health.

[69] Bui TD, Pham CK, Pham TH, Hoang LT, Nguyen TV, Vu TQ (2001). Cross-sectional study of sexual behaviour and knowledge about HIV among urban, rural, and minority residents in Viet Nam. Bull World Health Organ.

[70] Hung le Q, Vries PJ, Giao PT, Nam NV, Binh TQ, Chong MT (2002). Control of malaria: a successful experience from Viet Nam. Bull World Health Organ.

[71] Abe T, Honda S, Nakazawa S, Tuong TD, Thieu NQ, Hung le X (2009). Risk factors for malaria infection among ethnic minorities in Binh Phuoc, Vietnam. Southeast Asian J Trop Med Public Health.

[72] Peeters Grietens K, Xuan XN, Van Bortel W, Duc TN, Ribera JM, Ba Nhat T (2010). Low perception of malaria risk among the Ra-glai ethnic minority in south-central Vietnam: implications for forest malaria control. Malar J.

[73] Schratz A, Pineda MF, Reforma LG, Fox NM, Le Anh T, Tommaso Cavalli-Sforza L (2010). Neglected diseases and ethnic minorities in the Western Pacific Region exploring the links. Adv Parasitol.

[74] Verle P, Kongs A, De NV, Thieu NQ, Depraetere K, Kim HT (2003). Prevalence of intestinal parasitic infections in northern Vietnam. Trop Med Int Health.

[75] Uetani M, Jimba M, Kaku T, Ota K, Wakai S (2006). Oral health status of vulnerable groups in a village of the Central Highlands, southern Vietnam. Int J Dent Hyg.

[76] UNICEF, Viet Nam Administration of Preventive Medicine (2007). A summary of national baseline survey on environmental sanitation and hygiene situation in Viet Nam.

[77] Panter-Brick C, Clarke SE, Lomas H, Pinder M, Lindsay SW (2006). Culturally compelling strategies for behaviour change: a social ecology model and case study in malaria prevention. Soc Sci Med.

[78] Rheinlander T, Samuelsen H, Dalsgaard A, Konradsen F (2010). Hygiene and sanitation among ethnic minorities in Northern Vietnam: does government promotion match community priorities?. Soc Sci Med.

[79] Niemi M, Thanh HT, Tuan T, Falkenberg T (2010). Mental health priorities in Vietnam: a mixed-methods analysis. BMC Health Serv Res.

[80] World Bank (2009). Country social analysis – ethnicity and development in Vietnam. Summary Report.

[81] World Bank (2003). Vietnam – delivering on its promise.

[82] Axelson H, Sarah B, Pham DM, Ekman B, Gerdtham UG (2009). Health financing for the poor produces promising short-term effects on utilization and out-of-pocket expenditure: evidence from Vietnam. Int J Equity Health.

[83] Committee for Ethnic Minority Affairs, UNDP (2008). Final report – analysis of the P135-II Baseline Survey.

[84] UNFPA (2008). Reproductive health of ethnic minority groups in the greater Mekong sub-region.

[85] Wook CB (2003). Vietnamisation of southern Vietnam during the first half of the nineteenth century. Asian Ethnicity.

[86] Bonnin C (2010). Navigating fieldwork politics, practicalities and ethics in the upland borderlands of northern Vietnam. Asia Pac Viewp.

[87] Scott S, Miller F, Lloyd K (2006). Doing fieldwork in development geography: research culture and research spaces in Vietnam. Geogr Res.

[88] King M, Smith A, Gracey M (2009). Indigenous health part 2: the underlying causes of the health gap. Lancet.

[89] Gellner E Thought and change 1964.

[90] Stewart F (2003). Horizontal inequalities: a neglected dimension of development. Working Paper No. 1, Centre for Research on Inequality, Human Security and Ethnicity, (CRISE), Queen Elisabeth House.

[91] Solar O, Irwin A (2010). A conceptual framework for action on the social determinants of health.

[92] Sassi F, Carrier J, Weinberg J (2004). Affirmative action: the lessons for health care. BMJ.

